# Alcohol related cognitive impairments in schizophrenia patients : A case-control study

**DOI:** 10.1192/j.eurpsy.2023.734

**Published:** 2023-07-19

**Authors:** B. Angerville, N. Vaucher, F. Gierski, F. Benzerouk, S. Walesa, J. Seree, M.-C. Bralet, A. Benyamina, M. Naassila, A. Dervaux

**Affiliations:** 1 Filière universitaire d’addictologie, EPS Barthélemy Durand, Etampes; 2 UR PSYCOMADD, Université Paris saclay, Hopital paul Brousse, Villejuif; 3 Institut de Psychiatrie (GDR 3557), CNRS, Paris; 4 Service de Psychiatrie, Centre hospitalier Abbeville, Abbeville; 5 Cognition Health Society Laboratory (C2S - EA 6291), University of Reims Champagne Ardenne; 6 Department of Psychiatry, Marne Public Mental Health Institution & Reims University Hospital, Reims; 7 Crisalid department, EPSM Oise, Clermont-de-l’oise; 8 GRAP U 1247, INSERM-Université Picardie Jules Verne, Amiens, France

## Abstract

**Introduction:**

Cognitive impairment is a well-recognized key feature of schizophrenia. However, these cognitive impairments may be worsened by alcohol consumption. Up to 80% of patients with alcohol use disorders (AUD) display cognitive impairments. Screening for those impairments with a full neuropsychological assessment may be difficult. The Brief Evaluation Alcohol-Related Neuropsychological Impairments (BEARNI) is a specific tool for screening those impairments easy to implement in in clinical practice (Ritz et al. Alcoholism: Clinical and Experimental Research 2015; 39, 2249-60). To our knowledge, no previous studies have assessed the alcohol-related cognitive impairments using the BEARNI test in schizophrenia patients.

**Objectives:**

The objective of the study was to compare BEARNI mean scores between a group of schizophrenia patients with alcohol use disorders and a group of schizophrenia patients without alcohol use disorder.

**Methods:**

39 patients with schizophrenia and AUD (SCZ/AUD +) (82% males, mean age 44.9 ± 11.0 years-old) and 49 patients with schizophrenia without AUD (SCZ/AUD-) (65% males, mean age 42.6 ± 11.4 years-old) consecutively included in the study, were assessed using the BEARNI test. All patients met DSM-5 criteria for schizophrenia and AUD. Demographic and clinical variables were also collected, using the Alcohol Use Disorders Identification Test (AUDIT) and the Positive and Negative Syndrome Scale (PANSS). The primary endpoint of the study was the difference in BEARNI cognitive mean scores between the SCZ/AUD+ and SCZ/AUD- groups.

**Results:**

There was no difference between the two groups regarding demographic variables or PANSS mean scores (59.1 ± 12.8 vs 58.1 ± 14.0; t=-0.3; p=0.7). The AUDIT mean score was higher in the group of patients SCZ/AUD+ (20.6 ± 7.8 vs 1.6 ± 1.5; t=-14.7; p<0.0001). Total BEARNI and cognitive BEARNI mean scores were significantly lower in the group of patients SCZ/AUD+ compared to the group of patients SCZ/AUD- (10.6 ± 4.8 vs 12.6 ± 5.2; t=1.8, p=0.03 and 8.1 ± 3.9 vs 9.8 ± 3.6 t=2.0, p=0.04, respectively). The mean subscores of delayed verbal memory, alphabetical ordination, and alternating verbal fluency subtests were also significantly lower in the group of patients SCZ/+ group (respectively 1,0 ± 0.9 vs 1.6 ± 1.2, t= 2.5, p=0.01; 2,1 ± 1.2 vs 2.5 ± 1.1, t= 1.6, p=0.04; 3.3 ±1.5 vs 3.8 ± 1.5, t= 1.8, p=0.03).

**Conclusions:**

The present study found cognitive impairments using BEARNI test in schizophrenia patients with AUD compared to their counterparts without AUD. Screening alcohol related cognitive impairments using BEARNI could be easier in patients in schizophrenia patients with AUD than usual neurocognitive assessments

**Disclosure of Interest:**

None Declared

